# Genetic and life-history traits associated with the distribution of prophages in bacteria

**DOI:** 10.1038/ismej.2016.47

**Published:** 2016-03-25

**Authors:** Marie Touchon, Aude Bernheim, Eduardo PC Rocha

**Affiliations:** 1 Institut Pasteur, Microbial Evolutionary Genomics, Paris, France; 2 CNRS, UMR3525, Paris, France

## Abstract

Nearly half of the sequenced bacteria are lysogens and many of their prophages encode adaptive traits. Yet, the variables driving prophage distribution remain undetermined. We identified 2246 prophages in complete bacterial genomes to study the genetic and life-history traits associated with lysogeny. While optimal growth temperatures and average cell volumes were not associated with lysogeny, prophages were more frequent in pathogens and in bacteria with small minimal doubling times. Their frequency also increased with genome size, but only for genomes smaller than 6 Mb. The number of spacers in CRISPR-Cas systems and the frequency of type III systems were anticorrelated with prophage frequency, but lysogens were more likely to encode type I and type II systems. The minimal doubling time was the trait most correlated with lysogeny, followed by genome size and pathogenicity. We propose that bacteria with highly variable growth rates often encounter lower opportunity costs for lysogeny relative to lysis. These results contribute to explain the paucity of temperate phages in certain bacterial clades and of bacterial lysogens in certain environments. They suggest that genetic and life-history traits affect the contributions of temperate phages to bacterial genomes.

## Introduction

Temperate phages reproduce horizontally through a lytic cycle, like virulent phages, or vertically within a lysogenic host, as prophages (Lwoff, 1953). The lytic–lysogeny decision has presumably evolved from a trade-off between the relative opportunity costs of lysogeny (delayed lytic cycle) and lysis (low burst sizes under unfavorable conditions) (Weinbauer, 2004; Goldhill and Turner, 2014). In the lysogen, the interests of the prophages and their hosts are partly aligned because the former depend on the bacterium for replication. This may explain why some prophages protect the host from other phages, favor host growth or survival in certain environments, or encode toxins exploited for bacterial pathogenesis (McGrath *et al.*, 2002; Wagner and Waldor, 2002; Hyman and Abedon, 2010; Wang *et al.*, 2010). Temperate phages can thus shape the host evolution by affecting its population dynamics, through lysis, or by changing its gene repertoire, through lysogeny. They may also mediate horizontal gene transfer between bacteria (Jiang and Paul, 1998; Canchaya *et al.*, 2003a; Bobay *et al.*, 2013; Modi *et al.*, 2013).

The number of prophages in bacterial genomes is highly variable. Many bacteria are not lysogens, whereas some lysogens encode more than a dozen prophages (Fouts, 2006; Roux *et al.*, 2015). Genomic surveys showed that prophages are rare in small bacterial genomes (Casjens, 2003; Canchaya *et al.*, 2003b), where their frequency depends on the presence of restriction-modification systems (Oliveira *et al.*, 2014). To the best of our knowledge no other variables have been systematically associated with the distribution of prophages. The identification of such variables could provide new information on the genetic and life-history traits associated with lysogeny.

Environmental studies have shown that the frequency of lysogens varies in function of the environmental conditions. In particular, lysogens tend to be more abundant under conditions of low bacterial density, low nutrient concentration and low temperature (Cochran and Paul, 1998; Middelboe, 2000; Williamson *et al.*, 2002; McDaniel and Paul, 2005; Ghosh *et al.*, 2008; Pradeep Ram and Sime-Ngando, 2010; Shan *et al.*, 2014). Several arguments explain why these conditions favor lysogeny. First, they are associated with low concentrations of susceptible hosts, decreasing the benefits of lysis for the phage. Second, bacterial cells are smaller under poor growth conditions (Torrella and Morita, 1981; Akerlund *et al.*, 1995; Volkmer and Heinemann, 2011), providing fewer resources for the production of virions (reducing phage burst size). Third, prophage genes favoring host survival in poor growth conditions increase the fitness of lysogens over non-lysogens. These arguments suggest a tight association between bacterial growth conditions and lysogeny.

The frequency of prophages depends on the outcome of a series of processes, among which the frequency of infection, the probability of lysogenizaton and the rate of prophage loss (by induction or inactivation/deletion). Several experimental studies produced a detailed picture of the molecular mechanisms underlying these processes, especially in the interaction between *Escherichia coli* and the phage Lambda (reviewed in Ptashne, 1992). Defense systems, such as CRISPR-Cas and restriction-modification systems, protect bacteria from phages (Labrie *et al.*, 2010). The temperate phage that evades these defenses then faces the lytic–lysogeny decision. The frequency of lysogenization increases with the viral concentration inside the cell, which results from either high multiplicity of infection or small cell volume (Lieb, 1953; Kourilsky, 1973; Herskowitz and Hagen, 1980; St-Pierre and Endy, 2008). Finally, the rate of prophage loss by induction is higher in moments of decreased host viability, for example, following an SOS response (reviewed in Ptashne, 1992; Waldor and Friedman, 2005), under high temperatures (Bertani, 1954) or following loss of key bacterial regulators (Menouni *et al.*, 2013). These studies suggest that lysogeny is associated with a multitude of traits.

Both environmental and experimental studies showed that lysogeny is favored in smaller cells and under slow growth. Bacteria able to attain very short minimal doubling times under optimal conditions (fast growers) are poorly fit to grow under poor environmental conditions (Koch, 2001). The sizes of their populations in fluctuating environments change rapidly as a consequence of oscillations between high growth rates and rapid population collapses. It has been suggested that lysogeny represents a strategy of slow replication when the host provides few resources for reproduction in waiting for more propitious conditions for productive lysis (Stewart and Levin, 1984; Abedon, 2008). In this case, lysogeny should be more frequent among fast growers because they provide more variable resources for the production of virions. Bacteria with stable growth rates provide less variable resources for phage reproduction, decreasing the potential gains of lysogeny.

Here, we wished to gain some general understanding on the variables associated with lysogeny. For this, we analyzed three variables previously highlighted by environmental and genomic studies: (1) host genome size, as previously suggested (Casjens, 2003); (2) host pathogenicity, given the numerous prophage-encoded virulence factors found in bacterial pathogens (Brussow *et al.*, 2004; Abedon and Lejeune, 2005); (3) presence of CRISPR-Cas systems, given their role in defense against phages (Labrie *et al.*, 2010). We also analyzed two variables highlighted by experimental studies on *E. coli*: (4) average host cell volume, since larger *E. coli* cells favor lysis over lysogeny (St-Pierre and Endy, 2008) and (5) optimal growth temperature (OGT), since high temperature favors lysis (Bertani, 1954). We added a sixth variable, directly inspired from the above-mentioned theoretical arguments on the evolution of lysogeny (Stewart and Levin, 1984; Abedon, 2008). (6) Minimal doubling times under optimal conditions, since temperate phages infecting fast growers in moments of poor growth can increase their future burst size by lysogenization.

## Materials and methods

### Data on bacteria

We retrieved all 2110 complete bacterial genomes of 1196 species available in Genbank (ftp://ftp.ncbi.nih.gov/genomes/, last accessed in November 2013). We extracted from primary literature and from Vieira-Silva and Rocha (2010) the minimal doubling times (*d*) under optimal growth condition for 223 species of bacteria. OGTs were retrieved for 222 species from the DSMZ database (http://www.dsmz.de/microorganisms/) and from Vieira-Silva and Rocha (2010)). Mesophiles were defined as organisms with OGT over 15 °C and under 60 °C. In a complementary analysis we predicted the minimal doubling times (*d*_pred_) and the optimal growth temperatures (OGT_pred_) from the genomic sequences of each of the 1196 species using Growthpred with default parameters (Vieira-Silva and Rocha, 2010). The information related with the pathogenicity of bacterial species was taken from the literature (especially Brenner *et al.*, 2005).

### Analyses of phages

We retrieved the complete genomes of 831 phages from Genbank Genomes (last accessed in November 2013). Temperate phages were identified using PHACTS (McNair *et al.*, 2012). When the PHACTS probability score was not deemed confident we searched for the presence of integrases in phages using PFAM v26 (Finn *et al.*, 2008). More specifically, we searched for proteins with significant hits to the protein profiles for tyrosine (PF00589) and serine (PF07508 and PF00239) recombinase, using HMMER3 with default options (Eddy, 2011). These predictions were manually curated using the literature and the PhAnToMe database (http://www.phantome.org).

### Calculation of cell volume (*V*)

The volume of rods was determined from the average cell width (*W*) and length (*L*) using the formula for the volume of a cylinder capped by two hemispheres (Chrzanowski *et al.*, 1988): *V=* *π* (*W*/2)^2^(*L*−*W*)+(4/3) *π* (*W*/2)^3^. The volume of cocci was approximated by a sphere: *V*=(4/3) *π* (*W*/2)^3^. Length, width and shapes were retrieved from the literature (Brenner *et al.*, 2005).

### Detection of prophages

Prophages were detected in bacterial genomes using Phage Finder v4.6 (Fouts, 2006) (stringent option). We excluded all elements smaller than 18 kb, lacking matches to core phage proteins (e.g., terminase, capsid, head, tail proteins), or with more than 25% of insertion sequences. The latter were detected as in Touchon and Rocha (2007). Functionally related genes are usually grouped in one single region of the phage genome. Hence, elements containing several similar functional modules (e.g., integration, lysis, structural modules) more than 10 kb apart were considered as putative prophages coded in tandem. These few (~1%) elements were manually curated. Bacteria strains were considered as lysogenic when their genome contained at least one prophage. Bacterial species were defined as lysogenic when at least one strain was a lysogen.

### Detection of CRISPR-Cas systems

Clusters of *cas* genes were identified and classified using MacSyFinder (Abby *et al.*, 2014). CRISPR arrays were identified following a previously published methodology (Touchon *et al.*, 2011).

### Statistical analyses

Some of the variables used in this work were available for every strain (such as host genome size or the number of prophages), whereas others were only available for one or a few strains within a species (such as minimum doubling time). In 81% of the species only one complete genome was available. For the remaining species we either used all genomes (marked G in the figures) for comparisons between strain-specific traits or averaged strain-specific traits values across each species (marked S in the figures) for comparisons also involving species-specific traits. All major conclusions were controlled for the effect of phylogenetic dependency (see Supplementary Information and Supplementary Tables S1–S3). The data produced in this work is provided in Supplementary Dataset S1.

Associations between continuous variables were measured with the Spearman's rank correlation coefficient or (*ρ*) (Spearman, 1904). Associations between continuous and categorical variables were measured with the Wilcoxon rank-sum test (Wilcoxon, 1945). We analyzed the distribution of prophages with stepwise regressions. This standard statistical method consists in a stepwise integration of the different variables in the regression by decreasing order of contribution to the explanation of the variance of the data (Draper and Smith, 1998). We used the forward algorithm and the BIC criterion for model choice in the multiple stepwise regressions. The *P*-values associated with each variable were assessed using an *F*-test (Draper and Smith, 1998). We used JMP for the standard statistical analyses (Spearman, Wilcoxon and stepwise regressions) and the ape package in R for the analysis of phylogenetic dependency (Paradis *et al.*, 2004; see Supplementary Information).

## Results

### Identification and distribution of prophages in bacterial genomes

We searched for prophages in all available 2110 fully sequenced bacterial genomes (see Materials and methods). It was sometimes difficult to distinguish small partially degraded prophages from other mobile elements. Since the genomes of dsDNA self-transmissible temperate phages available in GenBank were all larger than 30 kb long, we restricted our search to prophages larger than 30 kb. We identified 2246 such elements. This constitutes our main data set of prophages. Most of these prophages encoded identifiable phage-specific functions such as integrases (86%), terminases (78%), tail- and baseplate-associated (79%), portal-associated (68%) and lysis-associated (66%) proteins. Hence, they are *bona fide* prophages.

We then searched for prophages between 18  and 30 kb long to assess how many prophage remnants or unknown small variants of intact prophages we have excluded. We identified 617 such elements. They encoded phage-specific functions at lower frequencies than in the main data set (resp. 51%, 38%, 62%, 25% and 34%), which might result from gene loss or errors in prophage identification. Unless explicitly stated otherwise, we present only the analyses made with the main data set of prophages, that is, the one including prophages larger than 30 kb. The results obtained in the analysis of the data set including smaller prophages (>18 kb) are qualitatively identical and can be found in Supplementary Material. To test if the prophages in the main data set were representative of the temperate phages present in GenBank we compared their sizes. The prophages were on average 48 kb long. This value was not significantly different from the average size of dsDNA temperate phages of GenBank (44.2 kb, see test statistics in Supplementary Figure S1). This suggests that our data set in unbiased in terms of prophage size.

**Figure 1:**
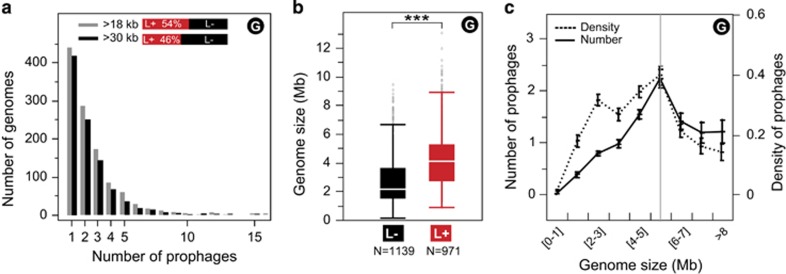
Distribution of prophages among all the genomes (G) used in the analysis. (**a**) Distribution of the number of prophages per genome in the two prophage data sets (>18 kb in gray, >30 kb in black). At the top: fraction of lysogens (L+) and non-lysogens (L−) in the two prophage data sets. (**b**) Box-plot of the distribution of size of the genomes (Mb) of non-lysogens (L−) and lysogens (L+) (***significant difference: *P*<10^−4^, Wilcoxon test). The horizontal white line at the center of the box plot represents the median. The bottom and top of the box represent the first and third quartiles. The external edges of the whiskers represent the inner 10th and 90th percentiles. (**c**) Distribution of the average number (full line) and density (dash line) of prophages per host genome in function of the size of the bacterial genome (Mb) (G). The vertical gray line separates small and average from the largest bacterial genomes. There is a significant positive association between the host genome size and the number of prophages in the former (Spearman's *ρ*=0.41, *P*<10^−4^) but not the latter (Spearman's *ρ*=−0.12, *P*>0.1). The association between the density of prophages and the host genome size is positive for the former (Spearman's *ρ*=0.35, *P*<10^−4^) and negative for the latter (Spearman's *ρ*=−0.21, *P*<10^−4^). Similar qualitative results were obtained in the analysis using the complementary data sets including smaller prophages and data averaged across species (Supplementary Figure S2).

Nearly half of the bacterial genomes contained at least one prophage (46% of lysogens; Figure 1a). While most lysogens had few prophages, some encoded up to 15 elements (Figure 1a). These and previous genomic (Casjens, 2003; Canchaya *et al.*, 2003b; Fouts, 2006; Roux *et al.*, 2015) and environmental analyses (Cochran and Paul, 1998; Ghosh *et al.*, 2008) suggest that lysogeny is very common in bacteria.

### The effect of the host genetic background on the frequency of lysogens

The median genome size of lysogens (4.1 Mb) was twice that of non-lysogens (2.4 Mb) (Figure 1b). We tested if this difference could be justified by the increase in bacterial genome size due to prophages. Prophages accounted for an average of 3.1% of the genomes of lysogens, with a maximum of 18% in *Bartonella tribocorum* CIP 105476. These values cannot justify the median genome size difference between lysogens and non-lysogens (1.7 Mb).

The observed association between bacterial genome size and lysogeny was non-monotonic. Firstly, we found a strong positive correlation between host genome size and the number and the density of prophages in genomes up to 6 Mb (Figure 1c). This association was not exclusively caused by the absence of prophages in the small genomes of obligatory endomutualists, since it remained valid in the range 3–6 Mb (lacking obligatory endomutualists). Secondly, bacteria with genomes larger than 6 Mb, which accounted for 12% of the species in our data set, showed no significant correlation between host genome size and the number of prophages. Instead, they showed a negative correlation between host genome size and prophage density (Figure 1c). It must be noted that most of these bacteria are lysogens (77%). Overall, these results show a strong positive association between bacterial genome size and the frequency of prophages in genomes smaller than 6 Mb and no association in the largest genomes.

Smaller bacterial genomes are more compact and have fewer accessory genes. This might lead to the selection of temperate phages with smaller genomes in these hosts. This does not seem to be the case, since we found no correlation between the average size of prophages and the host genome size (Spearman's *ρ*=0.01, *P*>0.8).

**Figure 2:**
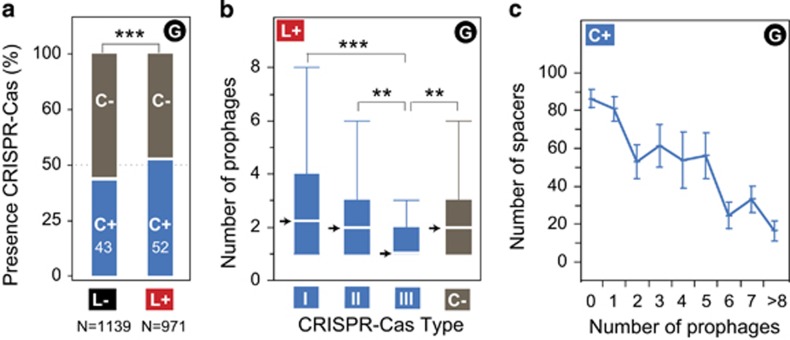
Analysis of the association between CRISPR-Cas systems and lysogeny among all the bacterial genomes (G). (**a**) Presence of CRISPR-Cas systems among lysogens (52%, L+) and non-lysogens (43%, L−) (***significant difference: *P*<10^−4^, *χ*2 test). (**b**) Distribution of the number of prophages per bacterial genome in lysogens (L+) in function of the presence of the different CRISPR-Cas systems (I, II, III) or when they are all absent (C−). Bacterial genomes encoding type III systems have fewer prophages than the others (***P<10^−4^ and **P<10^−3^, Wilcoxon test). Arrows indicate medians. (**c**) Distribution of the number of spacers in CRISPR arrays of bacterial genomes encoding CRISPR-Cas systems (C+) in function of the number of prophages per bacterial genome (Spearman's *ρ*=−0.21, *P*<10^−4^).

We analyzed the association between CRISPR-Cas systems and lysogeny (see Materials and methods). These systems were present in 47% of the genomes, which is consistent with previous estimates (Grissa *et al.*, 2007). Intriguingly, lysogens were more likely to encode CRISPR-Cas systems (Figure 2a). Among lysogens, the number of prophages was not correlated with the presence of these systems (*P*>0.6, Wilcoxon test). Type III CRISPR-Cas systems were relatively rare in the data set (8% of all the genomes). Contrary to the general trend, bacteria encoding these specific systems carried fewer prophages and were less likely to be lysogens than the others (Figure 2b and Supplementary Figure S3).

The number of spacers in CRISPR arrays is a measure of the number of sequences targeted by the system, and presumably of its capacity to provide protection against phages. Within genomes encoding CRISPR-Cas systems, lysogens had 30% fewer CRISPR spacers than non-lysogens (*P*<10^−4^, *χ*^2^ test). Furthermore, we found a negative association between the number of spacers in CRISPR arrays and the number of prophages in lysogens (Figure 2c). These results show a complex association between CRISPR-Cas systems and lysogeny: lysogens tend to encode CRISPR-Cas systems with small arrays of spacers, whereas non-lysogens are more likely to either lack these systems or encode long arrays of spacers. When all the genomes were put together, there was no association between the number of CRISPR-Cas spacers and the number of prophages (Spearman's *ρ*=0.04, *P*>0.1). As a result, this variable was not used in the multivariate analyses below.

### The effect of bacterial life-history traits on the frequency of lysogens

We tested the effect of bacterial life-history traits on the distribution of prophages. Most of these variables were only available at the species level, but 19% of the species in our data set were represented by more than one genome. We averaged the strain-specific data, such as genome size and number of prophages, across species (marked S in the figures). Initially, we restricted the analysis to species with published data on bacterial cell volume (139 species), pathogenicity (668 species), OGT (222) and minimal doubling time under optimal growth conditions (223). We could complement some of these analyses with computational predictions of the traits for the remaining species (see Materials and methods).

**Figure 3:**
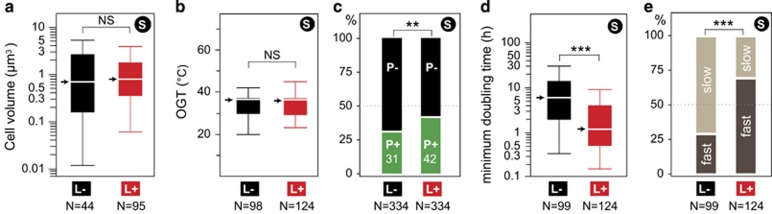
Analysis of the effect of species' (S) life-history traits on the distribution of lysogens. Box-plots of the distribution of the average cell volume (**a**) and optimal growth temperature (OGT, **b**) among the species with lysogens (red, L+) or lacking them (black, L−) (NS – nonsignificant differences: *P*>0.1, Wilcoxon test). (**c**) Proportion of species including bacterial pathogens (green, P+) or lacking them (black, P−) among species with lysogens (L+) or lacking them (L−) (**significant difference: *P*<10^−3^; *χ*^2^ test). Differences remained significant when controlling for genome size (*P*<10^−4^, stepwise regression) and phylogeny (*P*<10^−4^, generalized estimation equations analysis). (**d**) Box-plot of the distribution of the minimal doubling time under optimal conditions (*d*) among species with lysogens (L+) or lacking them (L−) (***significant difference: *P*<10^−4^, Wilcoxon test). Differences remained significant when controlling for bacterial genome size and phylogeny (*P*<10^−4^, generalized estimation equations analysis). (**e**) Proportion of fast (dark brown) and slow growers (light brown) among non-lysogens (L−) and lysogens (L+) (***significant difference: *P*<10^−4^, *χ*^2^ test). Arrows indicate medians.

Lysogens and non-lysogens showed no significant differences in the average cell volume (Figure 3a; see also Materials and methods). Among lysogens, we found no significant correlation between the average number of prophages carried by the genomes of a given species and the average volume of the corresponding cells after controlling for the host genome size (Supplementary Figure S4). These results show no evidence for an association between the average cell volume and lysogeny.

The OGT was not associated with lysogeny (Figure 3b, see Materials and methods). There was also no association between the average number of prophages and OGT among lysogens (Spearman's *ρ*=−0.06, *P*>0.5). The statistical power of this analysis is weak because 202 of the 222 species with known OGT were mesophiles. We increased the size of the data set by a factor of five by predicting OGT (OGT_pred_) for all the species. OGT can be predicted with high accuracy using protein sequences (Zeldovich *et al.*, 2007) (see Materials and methods). In this larger data set, the difference in OGT_pred_ between lysogens and non-lysogens remained nonsignificant when controlling for bacterial genome size (Supplementary Figure S5). Accordingly, the abundance of prophages was independent of OGT_pred_ among lysogens (Spearman's *ρ*=−0.007, *P*>0.8).

To test the association between virulence and the frequency of lysogens, we classed bacterial species into pathogens and non-pathogens (see Materials and methods). Such classifications are always coarse-grained descriptions of reality, since pathogenicity varies between strains, and depends on the eukaryotic host genetic background and physiological state. It is also difficult to class unambiguously some opportunistic bacteria (Pirofski and Casadevall, 2012). Nevertheless, species including pathogens were slightly more likely to contain prophages (see statistics in Figure 3c and Supplementary Figure S6). The observed difference might seem small, but pathogens in our data set have smaller genomes than the non-pathogens (*P*<0.03, median test). Accordingly, the frequency of prophages was higher in pathogens than in non-pathogens in all bins of genome size (see statistics in Supplementary Figure S6).

Finally, we tested the hypothesis that growth-related life-history traits affect the distribution of lysogens. We used the information on minimal doubling time under optimal conditions (*d*) to class bacterial species into fast growers (*d*<2.5 h) or slow growers (*d*⩾2.5 h), as previously suggested (Vieira-Silva and Rocha, 2010). Strikingly, we found that the minimal doubling time of lysogens was on average five times shorter than that of non-lysogens (Figure 3d). In fact, most bacterial species with lysogens were fast growers while most others were slow growers (Figure 3e). We found a weak and nonsignificant negative correlation between the average number of prophages in lysogens and their minimal doubling time (Spearman's *ρ*=−0.1, *P*>0.1). To test these conclusions in a larger data set, we predicted the minimal doubling time of the 1196 bacterial species used in this study with Growthpred (see Materials and methods). The negative association between the minimal doubling time and the average number of prophages per host genome was highly significant in this much larger data set (Spearman's *ρ*=−0.36, *P*<10^−4^), independently of host genome size (Supplementary Figure S7 and Supplementary Table S2).

### Multivariate analysis of the variables associated with lysogeny

**Figure 4:**
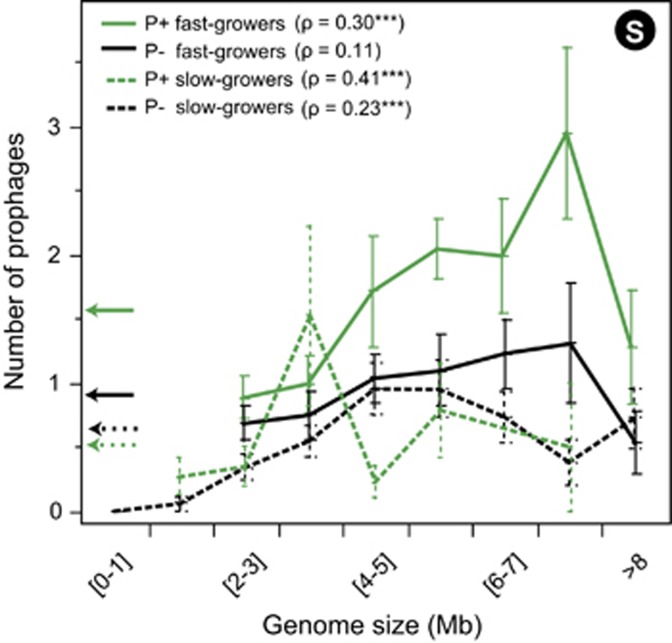
Distribution of the average number of prophages per bacterial genome in function of bacterial traits. The arrows on the left of the graph indicate the average number of prophages per genome (averaged across species) in each subset. The number of prophages per bacterial genome increases significantly with the host genome size in all cases (***P<10^−4^, the values of Spearman's *ρ* are reported for each analysis), except among non-pathogenic (P−) fast growers (Spearman's *ρ*=0.11, *P*>0.1).

We found significant associations between the frequency of lysogens and host genome size, pathogenicity, and minimal doubling time. These associations were partly independent. The significant association between minimal doubling time and the average number of prophages is observed among bacterial pathogens (Spearman's *ρ*=−0.48, *P*<10^−4^) and non-pathogens (Spearman's *ρ*=−0.22, *P*<10^−4^; Figure 4). The associations between the frequency of lysogens and both minimal doubling time and host genome size were strictly independent. We had previously shown that minimal doubling time and genome size do not correlate (Vieira-Silva and Rocha, 2010). In the present data set slow and fast growers had similar median genome sizes (Supplementary Figure S7, both ~3.3 Mb, *P*>0.8, median test). The analysis restricted to fast growers showed that pathogenic bacteria had more prophages than the others (*P*<10^−4^, Wilcoxon test), even if their genomes were of similar median size (*P*>0.5, median test).

We used stepwise multiple regressions to test the joint effects of the three variables and to identify which variables explained more of the variance in the distribution of prophages (see Materials and methods). All three variables contributed significantly for the statistical model (BIC criterion, Supplementary Table S4). The minimal doubling time accounted for most (66%) of the explained variance, followed by host genome size (23%) and pathogenicity (11%). We extended the stepwise regression analysis to measure the interaction terms between variables, but none passed the BIC criterion.

We showed above that bacterial genome size and the frequency of lysogens were correlated only for bacterial genomes smaller than 6 Mb (Figure 1). When we restricted our regression analysis to the bacterial genomes in this range of genome size, we obtained similar results (Supplementary Table S4). In this case, the minimal doubling time accounted for 63% of the explained variance.

The stepwise regression using all the data explained a small fraction of the variance (*R*^2^=0.14, *P*<10^−4^; Supplementary Table S4). This might be due to inaccuracies in the life-history traits data to the small number of prophages per genome (that affect the statistical power of linear models), and especially to epidemiological factors increasing intra-species variance. The life-history traits (for which phylogenetic studies are available) vary significantly only at large evolutionary scales (Galtier *et al.*, 1999; Vieira-Silva *et al.*, 2011). As a consequence, they might be more relevant to explain inter-species than intra-species variations in lysogeny. We tested if the inter-species variation was significant when accounting for intra-species variation, as suggested in Stearns (1977). To analyze the differences between species while reducing the effect of intra-species variation, we averaged the number of prophages per species in the set of 60 species for which there were at least five complete genomes. These species were represented by 718 genomes (34% of the data set). The stepwise regression using the 60 species showed an *R*^2^ of 0.41 (*P*<10^−4^; Supplementary Table S4), of which 78% was associated with the minimal doubling time. We varied the minimal number of genomes per species required to include a species in the analysis from 4 to 10 to test if this affected out conclusions. Our results show that this had little effect in the quality of the stepwise regression (Supplementary Figure S8).

The temperate phages of some bacterial phyla are poorly characterized. To test if this affected our study, we used stepwise regressions to analyze the data from Proteobacteria (which are 51% of all the bacterial genomes <6 Mb). This analysis also placed minimal doubling time as the most important explanatory variable, showing a switch in the relative order of the variables related with bacterial pathogenicity and genome size (Supplementary Table S4). Finally, we conducted the complementary analysis and removed Proteobacteria from the analysis. In this case the effects of minimal doubling time and the host genome size on the frequency of prophages remained significant (Supplementary Table S4), but the contribution of the pathogenicity was not significant. However, most large clades outside Proteobacteria had small genomes, fewer prophages and most species were non-pathogenic (Supplementary Table S5). This decreased the statistical power of the analysis.

## Discussion

The traits analyzed in this work explained over 40% of the variance between species when multiple genomes were available, but seemed to explain much less of the intra-species variation. Epidemiological variables, such as the environment where the strain was isolated, might be more appropriate to model the variation of the number of prophages within species. Several other factors may have affected our results, including the accuracy of prophage detection, the biased taxonomic characteristics of the genome reference data set and the quality of the data characterizing species' traits. These problems grow in importance when species are distant from well-studied model systems. For example, one of the three variables of the stepwise regression was no longer significant when we excluded the genomes from Proteobacteria from the analysis. Nevertheless, we found qualitatively similar trends, even if quantitatively different results, in our numerous controls, which included minimal size threshold for prophages, data acquisition (literature and computed data), phylogenetic dependency and restricted range of host genome size.

We found no significant association between the frequency of lysogens and the OGT or the average cell volume. Most phages infect a relatively narrow range of hosts that have similar traits in terms of OGT and average cell volume. The lytic–lysogeny decision evolves in response to the outcomes of previous host–phage infections in this range of hosts (Hyman and Abedon, 2010). It will evolve in function of temperature and cell size deviations relative to these absolute values, not the absolute values themselves, because these deviations provide information on the relative opportunity costs of lysogeny and lysis. Previous experimental works showed that lysogeny is shaped by the variability of prokaryotic physiology (Maurice *et al.*, 2013), and specifically that lysogeny is favored under suboptimal temperatures and in cells smaller than the species' average (Bertani, 1954; St-Pierre and Endy, 2008; Shan *et al.*, 2014). These deviations might drive some of observed intra-species variations in lysogeny.

CRISPR-Cas systems can prevent infections by phages when they carry spacers matching their sequences. This explains why genomes encoding systems with many spacers have fewer prophages, but not why bacteria with type I and type II systems are more likely to be lysogens. Recent studies have shown a poor correlation between the presence of these CRISPR-Cas systems and the rate of horizontal gene transfer (Touchon *et al.*, 2011; Gophna *et al.*, 2015). If CRISPR-Cas systems with few spacers are not actively involved in immune defense against phages, as previously proposed (Touchon and Rocha, 2010; Westra *et al.*, 2014), and if systems with many spacers actively protect bacteria from these elements, then our results can be reconciled with the previous experimental works: systems with long arrays prevent phage infection, resulting in few prophages in genomes, whereas the others have little impact on lysogeny.

While many lysogens encoded type I and type II CRISPR-Cas systems, very few encoded type III systems. Recent works suggested that type III-A CRISPR-Cas systems allow hosts to control their prophages (Goldberg *et al.*, 2014). Phages infecting bacteria carrying these systems might have evolved to avoid lysogeny, leading to the observed negative association between lysogeny and the presence of type III systems.

**Figure 5:**
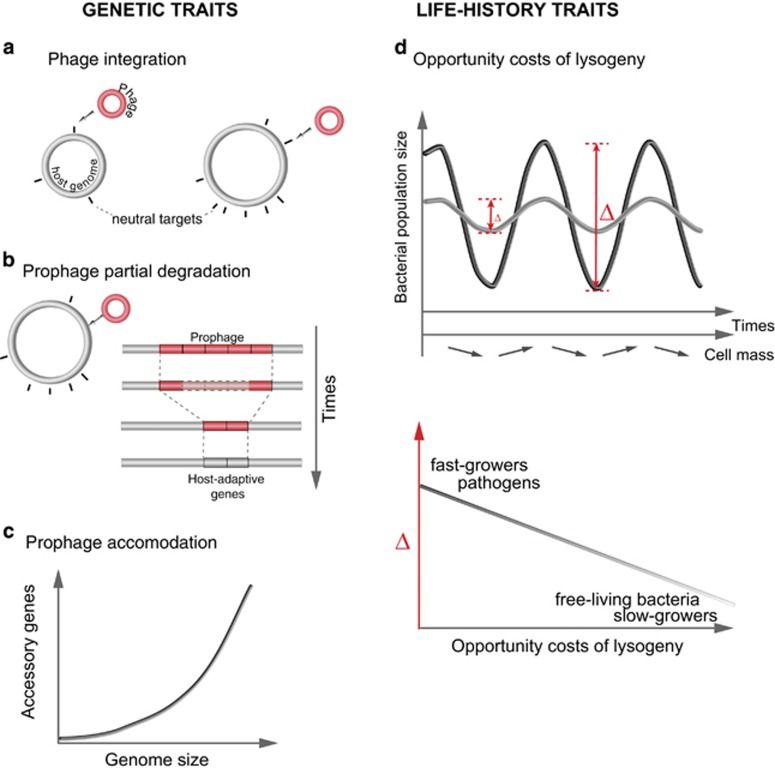
Genetic and life-history traits affecting the distribution of lysogens. (**a**) The number of neutral targets increases with the host genome size favoring phage integration. (**b**) Co-option of phage-related functions in degraded genetic elements increases with the number of prophages, and thus with the host genome size. After a certain time the few genes remaining in the bacterial genome may be too few or uncharacteristic to be detected as prophages. (**c**) Larger genomes have more accessory traits. (**d**) Fluctuating environmental conditions drive rapid expansion and contraction of bacterial populations (Δ), which are more important for fast growers and pathogenic bacteria than for slow growers and free-living bacteria (relative to pathogens with similar minimal doubling times). These fluctuations are associated with variations in cell mass and thus with burst size. They may also be associated with ecological conditions that constrain the lytic–lysogeny decision (such as the availability of susceptible hosts).

We confirmed that few small bacterial genomes are lysogens. We also observed that lysogens had much larger genome sizes than would be expected given the cumulated length of the prophages they contain. Why would larger genomes have more prophages? Larger genomes are expected to have more neutral targets for phage integration, facilitating the accumulation of these elements (Figure 5) (Bobay *et al.*, 2013). Larger genomes might directly result from the long-term accumulation of genes transferred by phages, for example, in lineages enduring frequent infections by phages. Yet, none of these hypotheses explains why this trend did not affect genomes larger than 6 Mb. If larger genomes resulted from intense selection for functional diversification by horizontal transfer, then selection for transfer might itself lead to mechanisms facilitating prophage acquisition (Cordero and Hogeweg, 2009; Smillie *et al.*, 2010). Selection for phage-related genes might saturate in the largest genomes because they contain many prophages. Alternatively, bacteria with many prophages might be very effective in preventing further phage infection (because prophages prevent infection by other phages), leading to the saturation of the number of prophages in larger genomes. Future work will be needed to quantify and disentangle the effects of host genome size on lysogeny and of lysogeny on host genome size.

We uncovered a strong negative association between minimal doubling times under optimal growth conditions and the frequency of lysogens. Minimal doubling times under optimal growth conditions and average doubling times across the diversity of conditions encountered by bacteria are not necessarily correlated (Boyce, 1984). Actually, the bacteria with the largest estimated effective population sizes are slow growers (Vieira-Silva *et al.*, 2011). The minimal doubling time is best interpreted as a key life-history trait associated with the r/K selection theory (Boyce, 1984) or with the choice between oligotrophic and copiotrophic lifestyles (Koch, 2001). Fast growers have population dynamics of alternating periods of feast and famine that are associated with large variations in growth rates and cell mass (Bremer and Dennis, 1996; Koch, 2001). The opportunity costs of lysogeny in these bacteria are very dependent on the host growth conditions at the time of infection (Figure 5). When environments are suitable, bacteria grow fast, the cell mass increases and populations are dense. This favors lytic over lysogenic cycles. Under conditions of slow bacterial growth, these populations remain at low densities and provide few resources for the production of virions; this favors lysogeny in waiting for more propitious conditions for the lytic cycle. The opportunity costs of lysogeny are generally less rewarding when phages infect slow growers because the host provides less variable resources for the production of virions. The ability to grow very fast under optimal conditions affects population dynamics (Koch, 2001), genome organization (Vieira-Silva and Rocha, 2010) and protein evolution (Vieira-Silva *et al.*, 2011). Our results suggest it also shapes the outcome of the interactions between bacteria and phages.

One could speculate that the low frequency of lysogens among slow growers could be caused by lower numbers of phages infecting these bacteria. In this case, virulent phages infecting slow-growing bacteria might also be rare. The little evidence available argues against this speculation, since many virulent phages of slow growers have been described in clades that lack lysogens in our analyses. For example, the population dynamics of cyanobacteria (slow growers and rarely lysogens) and other slow-growing marine heterotrophs are strongly affected by the numerous virulent viruses that infect them (Fuhrman, 1999; Wilhelm and Suttle, 1999; Winter *et al.*, 2010). There are also many virulent phages infecting clinical and environmental mycobacteria (Hatfull, 2010), all of which are slow growing according to our classification, but we identified few lysogens among them.

Our analyses suggest that lysogeny could be favored in bacterial pathogens. This could be explained by the virulence factors encoded by prophages (Wagner and Waldor, 2002; Brussow *et al.*, 2004), by the pathogens' peculiar cycles of population expansion and contraction (resembling those of fast growers, see above) and by the use of prophage induction as a biological weapon during colonization of a new niche (Bossi *et al.*, 2003; Gama *et al.*, 2013). The relative importance of these factors, if any, is not known.

Our work has shown associations between lysogeny and host genetic and life-history traits. These associations contribute to explain the rarity of prophages in certain clades, for example, those associated with small genomes or slow growth. Since prophages are one of the major sources of diversification of bacterial genomes, these traits may indirectly affect the evolvability of bacteria.
