# Supplementary material for: Genetic and life-history traits associated with the distribution of prophages in bacteria
Source: ISME J. 2016 Mar 25;10(11):2744–54. doi: 10.1038/ismej.2016.47 (PMC5113838; doi:10.1038/ismej.2016.47)
Supplement: Supplementary Information [file ismej201647x1.pdf]

Supplementary Information

Genetic and life-history traits associated with the distribution of prophages in bacteria

Touchon M[1,2], Bernheim A[1,2], Rocha EPC[1,2]

[1] Institut Pasteur, Microbial Evolutionary Genomics, Paris, 75015, France
[2] CNRS, UMR3525, Paris, 75015, France

SI Materials and Methods

Phylogenetic-independent contrasts. We estimated the phylogenetic signal of traits, which may spuriously inflate correlations between them, using Pagel's Lambda and Blomberg's K-statistic using the packages phytools and geiger for R (Harmon *et al*, 2008; Revell, 2012) and a 16SrRNA phylogenetic tree. We made a multiple alignment of the 16S sequences with MAFTT-v7.205, default parameters (Katoh and Toh, 2010). Poorly aligned regions were removed with BMGE using DNAPAM250 (Criscuolo and Gribaldo, 2010). Trees were computed by maximum likelihood with RAxML-v8 using the model GTRGAMMA (Stamatakis, 2014). Pairwise phylogenetic distances were computed from the distance matrix. Both measures revealed significant phylogenetic signal for all traits analyzed (Table S3). Therefore, we made independent contrast analyses to control for the association between continuous variables, and used generalized estimation equations to control for associations between continuous and discrete variable using the package ape (Paradis *et al*, 2004) in R. The analysis of contrasts showed in some clades some systematic outliers, caused by long internal branches in the tree. To include these points in the analysis without giving them unwarranted weight, we used nonparametric methods (Spearman rho) to examine the correlation between contrasts. All major statistical results remained significant after these controls (Table S1-S2).

Criscuolo A, Gribaldo S (2010). BMGE (Block Mapping and Gathering with Entropy): a new software for selection of phylogenetic informative regions from multiple sequence alignments. *BMC Evol Biol* **10:** 210.

Harmon LJ, Weir JT, Brock CD, Glor RE, Challenger W (2008). GEIGER: investigating evolutionary radiations. *Bioinformatics* **24:** 129-131.

Katoh K, Toh H (2010). Parallelization of the MAFFT multiple sequence alignment program. *Bioinformatics* **26:** 1899-1900.

Paradis E, Claude J, Strimmer K (2004). APE: Analyses of Phylogenetics and Evolution in R language. *Bioinformatics* **20:** 289-290.

Revell LJ (2012). phytools: an R package for phylogenetic comparative biology (and other things). *Methods in Ecology and Evolution* **3:** 217-223.

Stamatakis A (2014). RAxML version 8: a tool for phylogenetic analysis and post-analysis of large phylogenies. *Bioinformatics* **30:** 1312-1313.

Table S1- Control for phylogenetic dependence for the analysis done at the genomes level.

		Host genome size vs.	
		Number of prophages	Density of prophages
**Genomes N=2110**		Spearman's ρ = 0.37, $P<10^{-4}$	Spearman's ρ = 0.25, $P<10^{-4}$
	PIC analysis	Spearman's ρ = 0.28, $P<10^{-4}$	Spearman's ρ = 0.19, $P<10^{-4}$
	GEE analysis	$P<10^{-4}$	$P<10^{-4}$

Table S2- Control for phylogenetic dependence for the analysis done at the species level.

		Number of prophages vs.		
		Host genome size	Minimal doubling time (log)	Pathogenicity*
**Species N=223**		Spearman's ρ = 0.28, $P<10^{-4}$	Spearman's ρ = -0.46, $P<10^{-4}$	-
	PIC analysis	Spearman's ρ = 0.18, $P<0.007$	Spearman's ρ = -0.14, $P<10^{-4}$	-
	GEE analysis	$P<10^{-4}$	$P<10^{-4}$	$P<10^{-4}$

* N=668 species

Table S3- Estimation of the phylogenetic signal in the data.

		Pagel's Lambda	Blomberg et al.'s K	R-Function (package)
**Genomes**	Number of prophages	$\lambda = 0.62$, $P<3.10^{-159}$	$K = 1.10^{-5}$, $P<0.02$	phylosig (phytools)
	Density of prophages	$\lambda = 0.75$, $P< 4.10^{-171}$	$K= 1.10^{-5}$, $P<0.012$	phylosig (phytools)
	Host genome size	$\lambda = 0.99$, $P<1.10^{-100}$	$K = 1.10^{-4}$, $P<0.001$	phylosig (phytools)
**Species**	Number of prophages	$\lambda = 0.39$, $P<1.10^{-8}$	$K = 3.10^{-3}$, $P<0.2$	phylosig (phytools)
	Host genome size	$\lambda = 0.96$, $P<1.10^{-27}$	$K =0.3$, $P<0.001$	phylosig (phytools)
	Minimal doubling time (log)	$\lambda = 0.83$, $P< 3.10^{-31}$	$K= 0.02$, $P<0.002$	phylosig (phytools)
	Lysogens (Yes-No)	$\lambda = 0.96$		fitDiscrete (geiger)
	Pathogenicity (Yes-No)	$\lambda = 0.91$		fitDiscrete (geiger)

Table S4- Results of the stepwise regressions. Order represents the order of introduction of the variables in the stepwise regression (decreasing contribution to the R^2).

Regression of all data (N= 670)	order	estimate	Prob > F	cumulative R^2	% of the explained variance
log10 Minimal doubling time (h)	1	-0.771	$2.4*10^{-8}$	0.092	66%
Host genome size (Mb)	2	0.128	$3.19*10^{-6}$	0.124	23%
Pathogenicity	3	-0.164	0.00334	0.14	11%
CRISPR-Cas system	-	0	0.22 (NS)	-	-
Number of spacers	-	0	0.63 (NS)	-	-
intercept	-	0.792	1		

Genomes < 6 Mb (N=585)	order	estimate	Prob > F	cumulative R^2	% of the explained variance
log10 Minimal doubling time (h)	1	-0.642	$4.88*10^{-6}$	0.085	63%
Host genome size (Mb)	2	0.206	$5.55*10^{-7}$	0.122	28%
Pathogenicity	3	-0.148	0.0081	0.135	9%
intercept	-	0.491	1		

Only Proteobacteria < 6 Mb (N=298)	order	estimate	Prob > F	cumulative R^2	% of the explained variance
log10 Minimal doubling time (h)	1	-0.677	0.00877	0.096	71%
Host genome size (Mb)	3	0.214	0.00458	0.111	18%
Pathogenicity	2	-0.269	0.00348	0.135	11%
intercept	-	0.628	1		

Without Proteobacteria < 6 Mb (N=287)	order	estimate	Prob > F	cumulative R^2	% of the explained variance
log10 Minimal doubling time (h)	1	-0.729	$3*10^{-6}$	0.082	73%
Host genome size (Mb)	2	0.138	0.0029	0.113	27%
Pathogenicity	-	-0.055	0.39 (NS)	-	-
intercept	-	0.509	1		

Species with at least 5 complete genomes (N=60) **using the main dataset of prophages (>30 kb)**	order	estimate	Prob > F	cumulative R^2	% of the explained variance
log10 Minimal doubling time (h)	1	-1.137	$2*10^{-4}$	0.32	78%
Host genome size (Mb)	2	0.234	0.0055	0.41	22%
Pathogenicity	-	-0.042	0.77 (NS)	-	-
intercept	-	0.79	1		

Species with at least 5 complete genomes (N=60) **using the dataset of prophages (>18 kb)**	order	estimate	Prob > F	cumulative R^2	% of the explained variance
log10 Minimal doubling time (h)	1	-1.290	$7*10^{-4}$	0.30	77%
Host genome size (Mb)	2	0.286	0.0067	0.39	23%
Pathogenicity	-	-0.00001	0.99 (NS)	-	-
intercept	-	0.79	1		

Table S5- Datasets Characteristics

	Only Proteobacteria < 6 Mb	Without Proteobacteria < 6 Mb	Statistical Test
Number of species	298 (51%)	287 (49%)	
Median host genome size	3.9 Mb	2.8 Mb	Median test, $P<10^{-4}$
Number of lysogenic species	151 (51%)	131 (46%)	Chi2 test, NS, P>0.2
Number of pathogenic species	133 (45%)	90 (31%)	Chi2 test, $P<10^{-3}$
Average number of prophages/species	1.59 +/- 0.09	1.05 +/- 0.06	Chi2 test, P<0.05

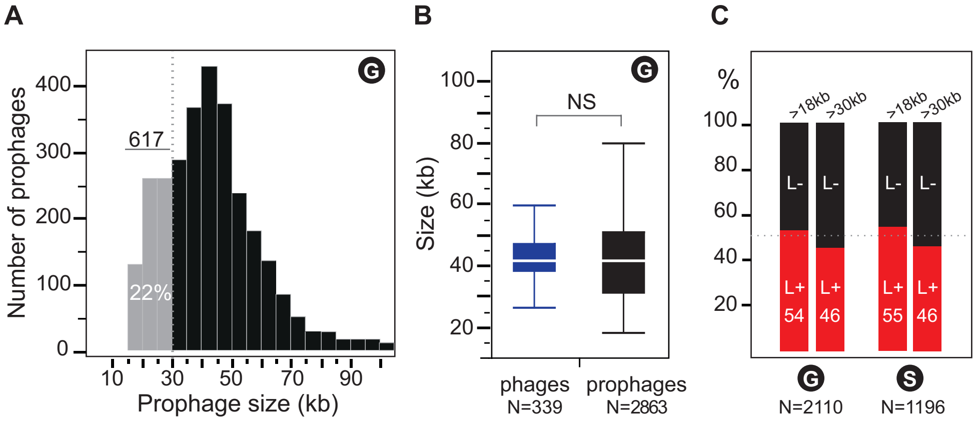

Figure S1. Characterisation of prophages. (A) Size distribution of small (18kb-30kb, grey), and large (>30kb, black) prophages. (B) Box-plot of the size distribution of GenBank's dsDNA temperate phages ("phages") and the prophages we detected in bacterial genomes ("prophages"). The center line of the box plot represents the median. The bottom and top of the box are the first and third quartiles. The external edges of the whiskers represent the inner 10th and 90th percentiles (NS: P>0.09, Wilcoxon test). (C) Fraction of lysogens (L+) and non-lysogens (L-) in all genomes (**G**) and averaged across species (**S**) for the two prophage datasets.

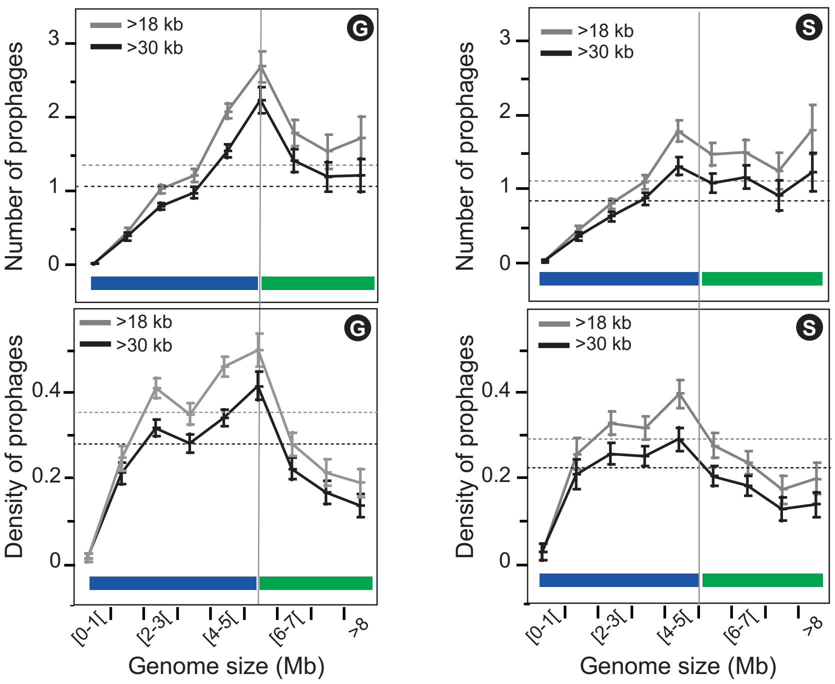

		(G) Number of prophages/Genome	(S) Number of prophages/Species
>18 kb	All	ρ = 0.43, $P<10^{-4}$	ρ = 0.35, $P<10^{-4}$
	<6Mb	ρ = 0.46, $P<10^{-4}$	ρ = 0.38, $P<10^{-4}$
	≥6Mb	ρ = -0.1, P>0.1	ρ = 0.03, P>0.6
>30 kb	All	ρ = 0.37, $P<10^{-4}$	ρ = 0.28, $P<10^{-4}$
	<6Mb	ρ = 0.41, $P<10^{-4}$	ρ = 0.33, $P<10^{-4}$
	≥6Mb	ρ = -0.12, P>0.1	ρ = 0.01, P>0.9

		(G) Density of prophages/Genome	(S) Density of prophages/Species
>18 kb	All	ρ = 0.28, $P<10^{-4}$	ρ = 0.17, $P<10^{-4}$
	<6Mb	ρ = 0.36, $P<10^{-4}$	ρ = 0.26, $P<10^{-4}$
	≥6Mb	ρ = -0.23, $P<10^{-4}$	ρ = -0.13, $P<10^{-2}$
>30 kb	All	ρ = 0.25, $P<10^{-4}$	ρ = 0.14, $P<10^{-4}$
	<6Mb	ρ = 0.35, $P<10^{-4}$	ρ = 0.23, $P<10^{-4}$
	≥6Mb	ρ = -0.21, $P<10^{-4}$	ρ = -0.1, P>0.1

Figure S2. Distribution of the number and density of prophages per genome in the four datasets: 2246 prophages larger than 30 kb (black), 2863 prophages larger than 18kb (grey), analysis done per bacterial genome (G), and analysis done using the average value per species (S). The vertical grey line separates smaller (blue) from larger genomes (green). The horizontal dash lines indicate the average of the average number (or density) of prophages in the two datasets of prophages. The Spearman's ρ association measures are indicated for each analysis.

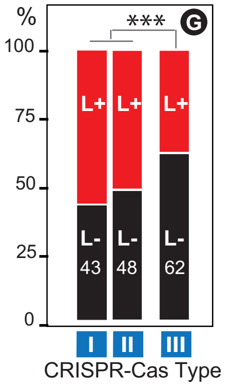

Figure S3. Proportion of lysogens (L+) and non-lysogens (L-) encoding CRISPR-Cas type I, II, III in the analysis using all genomes (G).

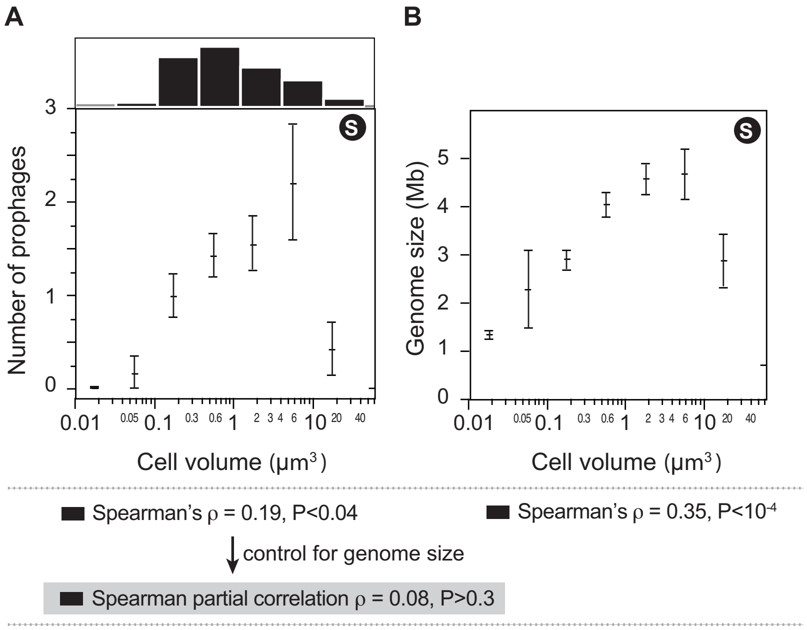

Figure S4. (A) Distribution of the average number of prophages per species (S) according to the volume of the host cell. The histogram on the top shows the distribution of the volume of the host cell. (B) Distribution of the average host genome size of each species (S) according to the cell volume. We indicate the values of Spearman's ρ for each analysis and the Spearman partial correlation once genome size is controlled for.

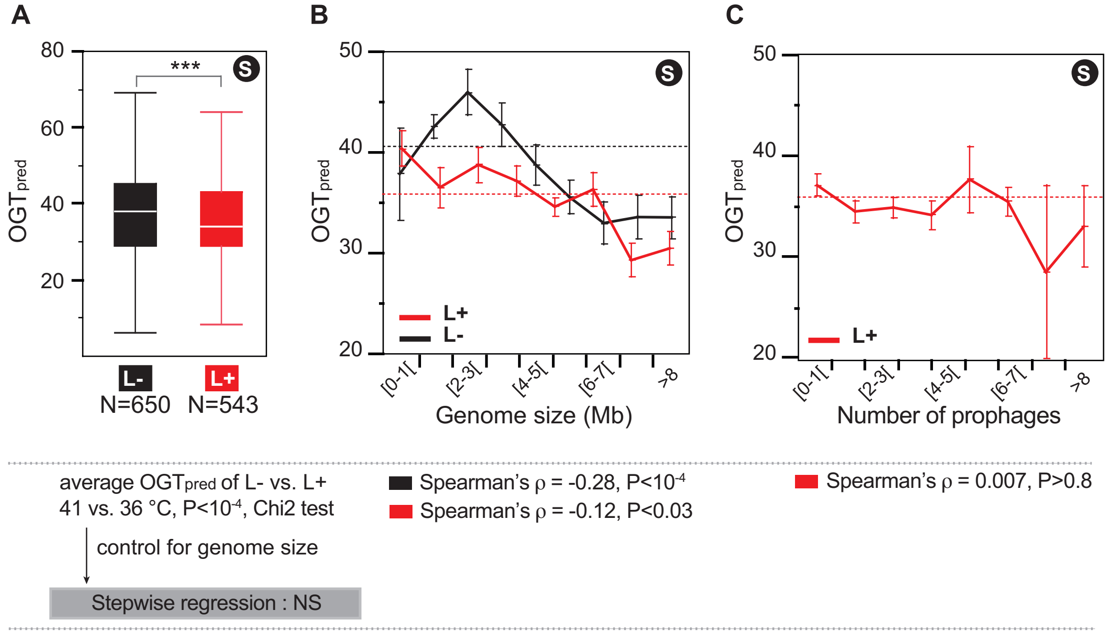

Figure S5. (A) The distributions of the predicted optimal growth temperatures (OGT_{pred}) of non-lysogens (L-) and lysogens (L+) are significantly different (P<10-4, Wilcoxon test). (B) Distribution of the OGT_{pred} in function of the genome size of non-lysogens (L-) and lysogens (L+). (C) Distribution of the OGT_{pred} in function of the number of prophages per genome in lysogens (L+). The black circle with an S indicates that these analyses were conducted in the dataset where genomes data are averaged across species. We indicate the values of the Spearman's ρ for each analysis and the result of the stepwise regression including genome size.

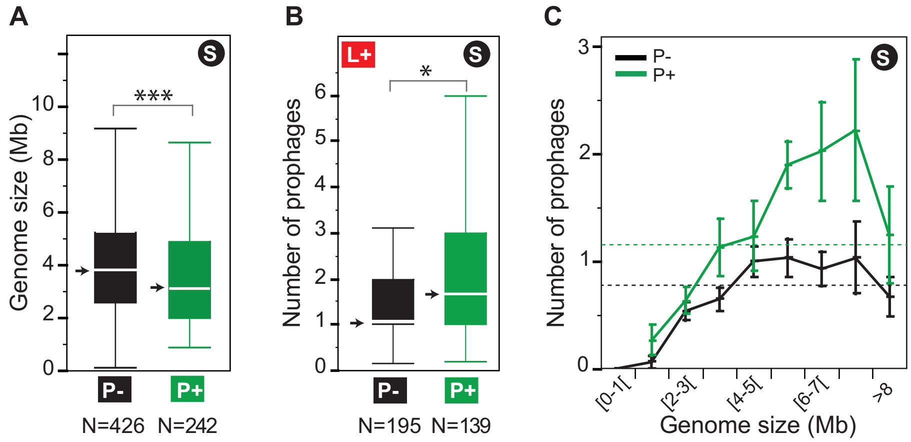

Figure S6. (A) Box-plot of the distribution of the genome size among non-pathogens (black, P-) and pathogens (green, P+). The medians indicated by arrows (3.8 and 3.1 Mb) are significantly different ($P<10^{-4}$, Wilcoxon test). (B) Distribution of the number of prophages among non-pathogens (P-) and pathogens (P+). The medians (arrows, 1 and 1.7) are significantly different (P<0.04, Wilcoxon test). (C) Distribution of the number of prophages according to the genome size of pathogens (P+) and non-pathogens (P-). The average number of prophages is higher in pathogens for every bin of host genome size (the bars in the figure represent the standard deviation of the average). The probability of this happening by chance is very low (P<0.0001, binomial test). The black circle with an S indicates that these analyses were conducted in the dataset where genomes are averaged across species. The top left red square with L+ indicates that the analysis is only made among lysogens.

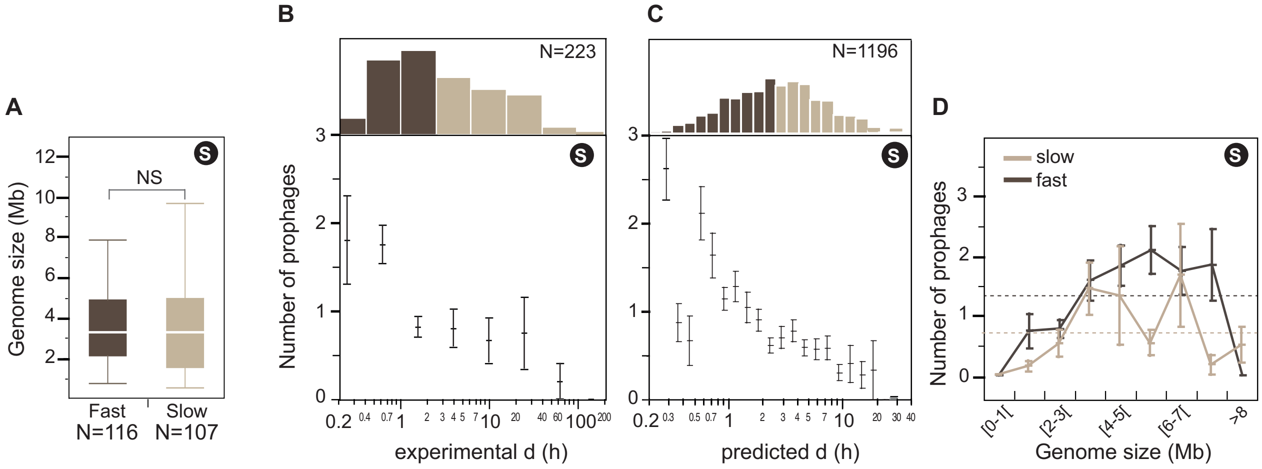

Figure S7. (A) Box-plot of the distribution of the host genome size among fast- (dark brown) and slow-growers (light brown). The medians are similar (both 3.3 Mb, NS, P>0.4, Wilcoxon test). (B) Distribution of the number of prophages per species according to the experimentally determined minimal doubling time (d) of the species. The association between the two variables is significant (Spearman's $\rho = -0.46$, $P<10^{-4}$). This correlation remains significant while controlling for genome size (Spearman partial correlation $\rho = -0.43$, $P<10^{-4}$, Wilcoxon test) and for phylogeny ($P<10^{-4}$, gee analysis). The histogram on the top shows the distribution of the the minimal doubling time of the species (C) Distribution of the average number of prophages per species according to the minimal doubling time of the species (predicted by Growthpred). This correlation remains significant while controlling for genome size (Spearman partial correlation $\rho = -0.31$, $P<10^{-4}$) and for phylogeny ($P<10^{-4}$, gee analysis). The histogram on the top shows the distribution of the minimal doubling time of the species. (D) Distribution of the number of prophages according to the host genome size of fast- (dark brown) and slow-growers (light brown). There is a significant positive correlation between host genome size and the number of prophages in fast- (Spearman's $\rho = 0.39$, $P<10^{-4}$) but also in slow-growers (Spearman's $\rho = 0.33$, $P<10^{-3}$). The black circle with an S indicates that these analyses were conducted in the dataset where genomes data are averaged across species.

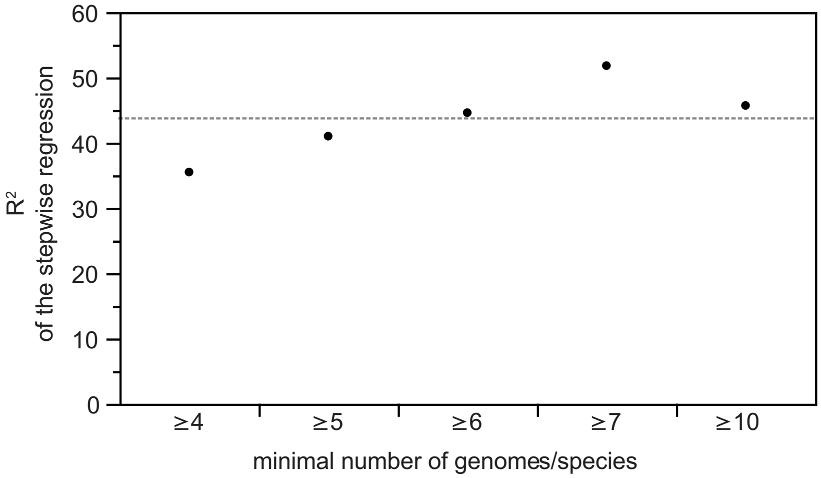

Figure S8. Variation of the R^2 of the stepwise regression with the minimal number of genomes per species required to include a species in the analysis. The range of R^2 variation is between 36% and 51% ($P<10^{-4}$).
